# Intelligent diagnosis of major depressive disorder with edge convolution and contrastive learning

**DOI:** 10.1371/journal.pone.0347870

**Published:** 2026-05-05

**Authors:** Dan Long, Chen Zhu, Lei Xiong, Zhou Long, Fangfang Dong

**Affiliations:** 1 Zhejiang Cancer Hospital, Hangzhou Institute of Medicine (HIM), Chinese Academy of Sciences, Hangzhou, Zhejiang, China; 2 School of Statistics and Data Science, Zhejiang Gongshang University, Hangzhou, Zhejiang, China; 3 The Second School of Medicine, Wenzhou Medical University, Wenzhou, Zhejiang, China; Zhejiang Normal University, CHINA

## Abstract

Based on functional connectivity (FC) matrices derived from resting-state functional magnetic resonance imaging (rs-fMRI) data, graph neural networks (GNNs), as an advanced deep learning technique, have been widely applied in major depressive disorder (MDD) diagnosis. However, conventional GNNs suffer from a critical limitation in preserving the spatial specificity of brain regions, which is attributed to their intrinsic node permutation invariance that ignores the unique order and specific roles of brain regions in neural circuits. To address this limitation, in this paper, we propose a novel deep learning framework, Graph Contrastive Learning based on Edge Convolution (EC-GCL), to analyze resting-state fMRI data from 1,160 participants, including 597 patients with MDD and 563 healthy controls. This framework integrates an edge convolution encoder, specifically designed to preserve the spatial specificity of brain regions, with a learnable graph augmentation module into an adversarial graph contrastive learning, thereby enhancing the extraction of discriminative MDD-related FC features and improving diagnostic classification accuracy. Compared with conventional machine learning and GNN models, our proposed EC-GCL achieved superior performance (AUC = 71.2%) and improved interpretability. In particular, the framework identified several key brain regions, including the dorsolateral superior frontal gyrus, thalamus, and insula, that are closely linked to the pathophysiology of MDD, which is consistent with the findings of prior neuroimaging studies. This study demonstrates that combining edge convolution with contrastive learning provides a robust and explainable method for MDD diagnosis. This provides new insights into depression and may support improvements in clinical practice.

## Introduction

Major depressive disorder (MDD) is a leading cause of disability worldwide, affecting more than 264 million individuals annually [1]. It is the second most common cause of death among young people aged 15–29 years. The diagnosis of MDD is typically based on systematic psychiatric evaluation in accordance with the criteria outlined in the Diagnostic and Statistical Manual of Mental Disorders, Fifth Edition (DSM-5), combined with an assessment of patients’ clinical symptoms and medical history. However, the consistency of such subjective diagnostic procedures remains unsatisfactorily low, with a reliability coefficient as low as 0.25 [2]. Consequently, there is an urgent need for objective approaches to improve the accuracy of MDD identification.

Neuroimaging studies have revealed that MDD patients exhibit abnormalities in both gray matter (e.g., frontal lobe, hippocampus, amygdala) and white matter (e.g., thalamus, striatum, associated fiber bundles) [3–5]. These subtle structural changes, if captured effectively, could serve as biomarkers for MDD diagnosis and provide new perspectives for intelligent clinical assessment. But they are difficult to identify. To address this challenge, researchers aim to leverage advanced machine learning techniques to extract neuroimaging biomarkers from multi-modal data, including structural magnetic resonance imaging (sMRI), functional MRI(fMRI), and diffusion tensor imaging (DTI), helping diagnose and evaluating treatment for depression [6]. Despite these efforts, the core pathophysiological mechanisms of MDD remain poorly understood due to its high complexity. In recent years, resting-state fMRI (rs-fMRI) has been proven to be more effective than structural imaging or task-based fMRI in MDD classification [7]. The studies revealed that there is a strong correlation between the brain’s microscopic neural circuits and the macroscopic rs-fMRI functional network [8]. Furthermore, rs-fMRI derived functional connectivity(FC) patterns between the limbic system and frontal-striatal circuits can be used to define different neurophysiological subtypes of MDD [9]; these subtypes correlate with clinical symptom profiles and can predict responsiveness to neurostimulation therapies, further confirming the potential of fMRI-based brain networks as rich sources of diagnostic biomarkers. In short, a lot of many studies have shown that rs-fMRI is well-suited for thoroughly describing brain network features, which makes it a potential objective way for MDD diagnosis.

In the past few years, great progress has been made in computer-aided diagnosis for brain disorders using rs-fMRI data. Since brain networks inherently exhibit a graph structure, with brain regions as nodes and functional connections as edges, graph neural networks (GNNs) have been widely used to classify brain networks from fMRI scans. Prior studies have demonstrated that GNN-based models outperform traditional machine learning methods such as Multilayer Perceptron (MLP) in predicting depression severity [10]. Our previous research integrated global network attribute features into GNN, which also confirms this view [11]. However, traditional GNNs often miss spatial details about brain regions. Their message-passing mechanisms treat all nodes indiscriminately, ignoring the unique order and specific roles of brain regions in neural circuits, which leads to a contradiction between GNNs' node permutation invariance and brain region specificity. This limitation reduces both classification accuracy and interpretability. To address this issue, researchers have attempted to add special features to each node when building brain networks [12]. Unfortunately, these modifications do not completely resolve the problem of indiscriminate node treatment during message passing. Consequently, classification performance remains suboptimal.

In this study, we present a new framework that preserves the spatial specificity of nodes with edge convolution instead of traditional graph convolution, capturing directional connection patterns. This method better reflects the unique functions of neural circuits. Furthermore, to make the model stronger and less dependent on labeled data, we use a contrastive learning method based on adversarial ideas. By generating augmented graphs through automatically learning edge deletions while maintaining classification consistency, the model achieves effective data augmentation and enhances interpretability. This design not only strengthens feature representation but also enables the identification of key brain regions closely associated with the pathophysiology of MDD.

## Method

All data came from the REST-Meta-MDD project (http://rfmri.org/REST-meta-MDD), which includes fMRI scans from 2,428 participants across 17 hospitals in China. To meet the requirements of the study, we further screened the dataset by following the same procedures described in our previous work [13]. After screening, a total of 1,160 subjects from 10 independent sites were retained, including 597 with MDD and 563 healthy controls. Then, the data for each subject were preprocessed as described in Yan et al. without global signal regression [14]. Specifically, the preprocessing of these fMRI data was performed using Data Processing Assistant for Resting-State fMRI (DPARSF) based on SPM12 in MATLAB 2018a. Following our previous study [11], by dividing the brain into 116 regions of interest (ROIs) with the AAL atlas, we adopted ROI-based FC analysis: the brain networks were built by calculating FC between regions with Pearson correlation, and setting the diagonal elements of the connectivity matrix to zero [15].

### Architecture overview

In this paper, an EC-GCL framework is proposed for MDD diagnosis, which is an adversarial graph contrastive learning framework with learnable data augmentation based on 1D convolution (edge convolution) operations. The framework is mainly composed of a graph augmentation module, two classifier branches for original data and augmented data. Each classifier branch has the same architecture but non-shared parameters, including a feature encoder, a projection head, and a classifier. The specific process is as follows: Firstly, we extract the FC matrix from fMRI as the input graph *X*, and the graph *X* is fed into a graph augmenter to obtain an augmented graph $X^{\prime }$. Then, the original graph *X* and the augmented graph $X^{\prime }$ are fed into the dual branch network with the same encoder ℰ, the same projector 𝒫 and the same classifier 𝒞, respectively, to obtain the contrastive embedding $\left(z,z^{\prime }\right)$ and classification result $\left(\hat{y},{\hat{y}}^{\prime }\right)$. Finally, the model is trained by the total loss function with contrastive loss and classification loss to optimize the feature extraction and classification performance.

The entire forward network can be formulated as:


z=𝒫(ℰ(X)), (1)

$$z^{\prime }=𝒫(ℰ(X^{\prime })),$$ (2)

$$\hat{y}=𝒞(ℰ(X)),$$ (3)

$${\hat{y}}^{\prime }=𝒞(ℰ(X^{\prime })).$$ (4)

In this network, the encoder ℰ sequentially consists of the following components: two edge convolutional layers, a 1 × *R* convolutional layer, an *R* × 1 convolutional layer, and a linear layer. To introduce the non-linearity, an activation function is applied after each component. Thus, the encoder can be formulated as:


$$E(H)=\sigma (Econv_{2}(\sigma (Econv_{1}(H)))),$$ (5)

$$ℰ(H)=\sigma (\sigma (\sigma (E(H)*w_{1\times R})*w_{R\times 1})W_{1}+b_{1}),$$ (6)

where *H* represents the input feature, *Econv* represents the edge convolution, σ represents the activation function, *w*_*R*×1_ and *w*_1×*R*_ are the weights of R × 1 convolution and 1 × R convolution layers, and *W*_1_ and *b*_1_ are weights of linear layer.

Both the projector head 𝒫 and classifier 𝒞 are two different MLPs with onehidden layer:


$$𝒫(H)=\sigma (HW_{2}+b_{2})W_{3}+b_{3},$$ (7)

$$𝒞(H)=\sigma (HW_{4}+b_{4})W_{5}+b_{5}.$$ (8)

**Fig 1:**
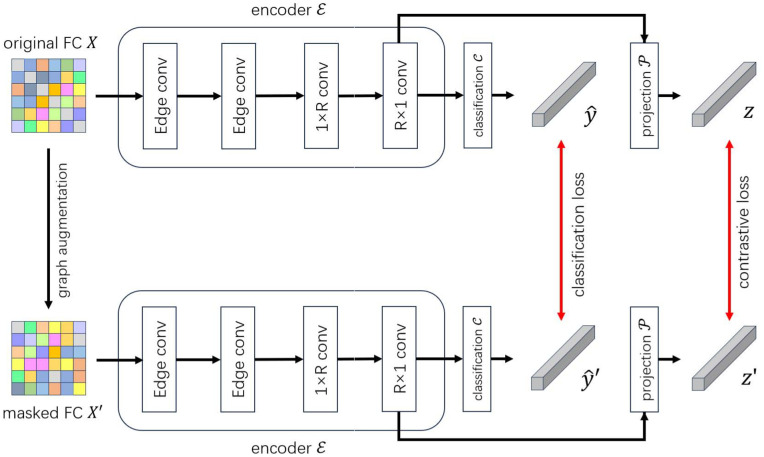
The overall architecture.

The details are shown in Fig 1. Note that the activation layers and linear layers are not shown in the figure for simplicity.

### Edge convolution operation

To avoid the permutation invariance problem of GNN, we propose to replace graph convolution with edge convolution blocks. Specifically, for the feature map of the *l*-th layer $X^{l}\in {\mathbb{R}}^{R\times R\times M}$, where *R* is the number of regions of interest and *M* is the input channel, a 1 × *R* convolution filter is used to get $c^{l}\in {\mathbb{R}}^{R\times 1\times N}$, and an *R* × 1 convolution filter is used to get $r^{l}\in {\mathbb{R}}^{1\times R\times N}$, where *N* is the output channel. Then, the edge convolutional block can be defined as:


$$c^{l}=X^{l}*w_{1\times R}^{l}+b_{c}^{l},$$ (9)

$$r^{l}=X^{l}*w_{R\times 1}^{l}+b_{r}^{l},$$ (10)

$$X^{out}=Econv(X^{l})=concatenate(c^{l}⊗1^{T},1⊗r^{l}),$$ (11)

where * represents the convolutional operation, $w_{1\times R}^{l}$ and $b_{c}^{l}$ represent the weight and bias of the 1 × *R* convolutional filter, $w_{R\times 1}^{l}$ and $b_{r}^{l}$ represents the weight and bias of the *R* × 1 convolutional filter, ⊗ represents the outer product, and $1\in {\mathbb{R}}^{R\times 1}$ is an *R*-dimensional vector containing a single value of 1, while $X^{out}\in {\mathbb{R}}^{R\times R\times 2N}$ is the output of the edge convolution operation.

**Fig 2:**
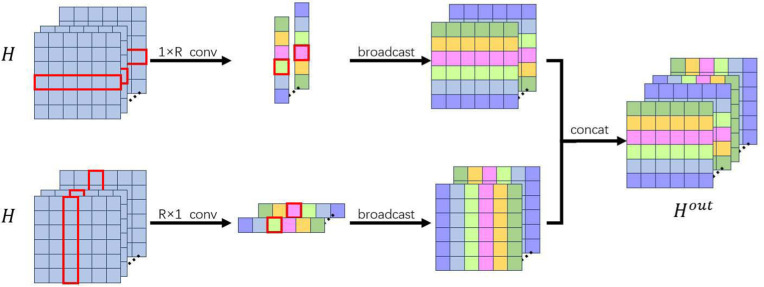
Flow of edge convolution (Edge conv).

By using edge convolution, the framework captures connections between nodes through specific filters with learnable weights, while maintaining the inherent specificity of the nodes. Specifically, the input FC matrix is processed by two one-dimensional convolutional operations: (1) The 1 × *R* convolutional filtering is used to focus on the brain region features in the “row” dimension, capturing the connection pattern between the current brain region and all other brain regions; (2) The *R* × 1 convolutional filtering is used to focus on the brain region features in the “column” dimension, capturing the connection pattern between all other brain regions and the current brain region. The results of the two operations are concatenated to form a feature representation, which not only contains the connection information between nodes, but also retains the spatial specificity of a single node (brain region), avoiding the limitation of “indiscriminate treatment of nodes” in traditional graph convolution algorithms. The detailed edge convolution algorithm is shown in Fig 2.

### Graph augmentation

**Fig 3:**
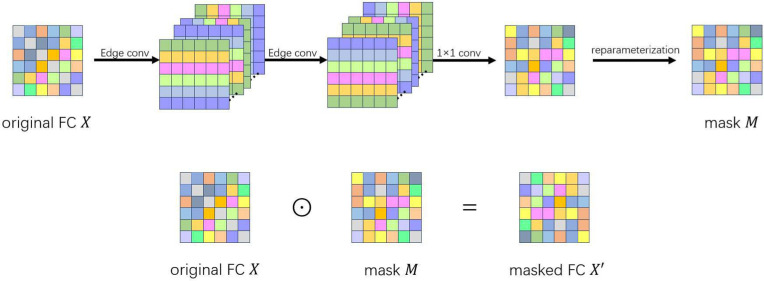
The graph augmentation.

The graph augmentation module aims to generate a novel data view by masking edges of the original FC matrix, and meanwhile preserve edges that are informative for the classification task and ensure that the features derived from the network remain as invariant as possible. Unlike the random edge deletion operations, in this paper, we present a learnable graph augmentation method based on contrastive learning. To be specific, the new graph is produced by training an edge drop mask from a probability matrix. The probability matrix reflects the importance of edge pairs for classification. The specific process is shown in Fig 3.

Given the input FC matrix $X\in {\mathbb{R}}^{R\times R}$, we feed it into the encoder 𝒢 to get an edge-dropping probability matrix $P\in {\mathbb{R}}^{R\times R}$. The encoder 𝒢 contains two edge convolutional layers with nonlinear activations, a 1 × 1 convolution operation, and a sigmoid activation, as shown in Fig 3. The process can be defined as:


$$H^{1}=\sigma (Econv_{3}(X)),$$ (12)

$$H^{2}=\sigma (Econv_{4}(H^{1})),$$ (13)

$$P=𝒢(X)=Sigmoid(H^{2}*w_{1\times 1}),$$ (14)

where *Econv*_3_ and *Econv*_4_ are two edge convolutional operations with different weights, which are non-shared with the weights of edge convolution layers in the main encoder ℰ; $\sigma (\dot{)}$ denotes the nonlinear activation function, *H*^*l*^ and *H*^2^ represent the hidden represented features, and *w*_1×1_ means the weight of the 1 × 1 convolutional filter. After the feature extraction for the original FC matrix, by a 1 × 1 convolution, the channel number of features *H*^2^ is reduced to 1, and then a sigmoid function is applied to normalize the element values to the range [0, 1], yielding a probability matrix that denotes the masking probability of each edge in the original FC matrix. In this way, the FC matrix is transformed into an edge-dropping probability matrix.

To obtain an edge-dropping graph, a binary mask is required. Then, we sample a mask matrix $M\in (0,1{)}^{R\times R}$ from a Bernoulli distribution parameterized by the edge dropping probability matrix *P*(i.e., *M* ∼ *Bernoulli*(*P*)), where 0 indicates that the corresponding edge is completely masked and discarded, and 1 indicates that the edge is retained. However, this sampling process results in the gradient not being able to back-propagate. To enable gradients to propagate backwards, we use the following re-parameterization trick to ensure the graph augmenter is effectively trained:


$$M=Sigmoid((\log\frac{E}{1-E}+\log\frac{P}{1-P})/\tau_{1})$$ (15)

where $E\in {\mathbb{R}}^{R\times R}$ is constructed based on *E*_*ij*_ ∼ Uniform(0,1), and $\tau_{1}$ represents a temperature parameter that controls the smoothness of the re-parameterized sampling functions. As $\tau_{1}\to 0$, *M* gets closer to the sampled binary mask.

After obtaining the edge dropping mask *M*, we apply it to the original FC matrix *X* by element-wise multiplication to obtain the augmented graph $X^{\prime }$:


$$X^{\prime }=M⊙X.$$ (16)

### Loss functions

To train the feature extractor and graph augmenter, we use a loss function that forces the two feature vectors of the positive sample pair to be close and the two feature vectors of the negative sample pair to move away. Specifically, the original FC matrix *X* and the augmented graph $X^{\prime }$ derived from it will be considered as a positive pair, while those from different original FC matrices are negative pairs. In this way, the trained feature extractor can capture the most important information while removing the redundant. Specifically, the contrastive loss is defined as follows:


$$L_{con}=-\frac{1}{B}\sum_{i=1}^{B}\log\frac{exp(sim(z_{i},z_{i}^{\prime })/\tau_{2})}{\sum_{j=1}^{B}exp(sim(z_{i},z_{j}^{\prime })/\tau_{2})},$$ (17)

where *B* is the number of graphs in the batch, $\tau_{2}$ is a temperature hyper-parameter, and *sim*() is the cosine similarity metric, i.e.,


$$sim(z_{1},z_{2})=\frac{z_{1}^{T}z_{2}}{\left\|z_{1}\|\|z_{2}\right\|}.$$ (18)

However, this contrastive loss can be easily minimized by discarding the smallest number of edges. To encourage the graph augmenter to drop more edges, we use a regularization function to regularize the ratio of discarded edges. The regularization loss function is defined as the mean value of all elements in the edge-dropping mask $M_{i}$:


$$L_{reg}=\frac{1}{BR^{2}}\sum_{i=1}^{B}1^{T}M_{i}1.$$ (19)

To train the feature extractor and classifier, we use a classification loss function simultaneously with the contrastive loss. For the classifier, we get $\hat{y}$, which is the classification result of the original FC matrix *X*, and ${\hat{y}}^{\prime }$, which is the classification result of the augmented matrix $X^{\prime }$. Their average cross-entropy is used for the final classification loss:


$$L_{cls}=-\frac{1}{B}\sum_{i=1}^{B}y_{i}\log\frac{{\hat{y}}_{i}+\overset{\prime }{\underset{i}{\hat{y}}}}{2},$$ (20)

where *y* is the label of the corresponding samples *X* and $X^{\prime }$.

Finally, the total loss function of our framework is expressed as:


$$L_{total}=L_{con}+\lambda_{1}L_{cls}+\lambda_{2}L_{reg},$$ (21)

where $\lambda_{1}$ and $\lambda_{2}$ are hyper-parameters, which balance the three loss functions. In Eq (21), the unknown variables are the model parameters to be optimized, including the weights and biases of the edge convolutional layers, linear layers, and convolutional layers in the encoder ℰ, encoder 𝒢, projector 𝒫, and classifier 𝒞.

## Experiments and results

### Experimental setup

Our algorithm is implemented on an NVIDIA 4060 GPU with Python 3.9.16 and PyTorch 2.0.0. The overall network is optimized using the Adam optimizer with default momentum parameters. To maintain the stability of the training, we set up a separate optimizer for the graph augmentation module during the training process, with its learning rate being smaller than that of other parts. The learning rate of the graph augmentation is *lr*_1_ = 1*e* − 4, and the others are *lr*_2_ = 1*e* − 3. The total number of epochs is set to 300 and the batch size is 16. The hyper-parameters in the loss function are $\lambda_{1}=1.0$, $\lambda_{2}=0.2$ (which is determined by grid search method). The activation function in graph augmentation 𝒢 and feature encoder ℰ is LeakyReLU (*negative*_*slope*_ = 0.33), and the activation function in the projection head 𝒫 and the classifier 𝒞 is ReLU. The specific experimental parameters are shown in Table 1.

**Table 1:** Experimental parameter settings.

Parameter name	Parameter description	value
epochs	The number of iterations when training	300
batch_size	The batch size of training data	16
$\lambda_{1}$	The coefficient of the classification loss	1.0
$\lambda_{2}$	The coefficient of the regularization term	0.2
*lr*_1_	Learning rate of data augmentation module	0.0001
*lr*_2_	Learning rate for other parts of the model	0.001
$\tau_{1}$	The parameterized temperature in Bernoulli distribution	1.0
$\tau_{2}$	The temperature parameter in contrastive loss	1.0
view_hidden_d	The number of channels in data augmentation module for hidden layers	16
edge_dim	The number of channels in the encoder before the first dimensionality reduction	8
node_dim	The number of channels in the encoder after the first dimensionality reduction	16
out_dim	The dimension of the encoder’s output features	64
proj_dim	The dimension of projector’s output features	128

**Fig 4:**
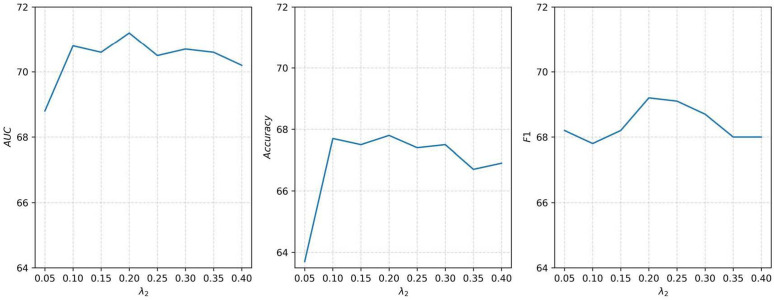
Effects of different regularization coefficients $\lambda_{2}\;$ on classification performance.

The regularization coefficient $\lambda_{2}$ in the total loss function is crucial to control the edge masking intensity for the original graph in the data augmentation module, which in turn affects the model’s classification performance. To identify the optimal value of $\lambda_{2}$, we employed a grid search strategy, evaluating coefficients within the range of 0.05 to 0.40. As presented in Fig 4, the experimental results demonstrate that the model attains its peak performance when $\lambda_{2}$ is set to 0.2.

To evaluate the computational efficiency of the EC-GCL framework, we recorded the total number of parameters, training time, and inference time. The total number of trainable parameters of the EC-GCL framework is 0.207 M. The average training time per epoch was 3.57 seconds, with a total training time of approximately 18 minutes for 300 epochs. For inference, the average time to process a single sample was 5.88 milliseconds(ms). This computational efficiency, together with its superior diagnostic performance (presented in subsequent sections), indicates that the framework achieves a favorable trade-off between accuracy and efficiency.

In addition to achieving accurate classification, it is also crucial to explore the relationship between disease and the brain. A natural advantage is that our method allows for interpretability analysis. In the contrastive learning framework, we discard edges that are not useful for classification through a learnable graph enhancement method, preserving functional connections (edges) that are important for disease classification. By analyzing these retained edges, we further calculate the importance of brain regions, thereby identifying the top ten brain regions most relevant to classification.

Specifically, we calculate the importance score by the edge-dropping mask *M* as follows:


$$S=sum_{row}(M+M^{T}),$$ (22)

where *sum*_*row*_ represents row-wise summation of the matrix *M*. By adding the edge-dropping matrix *M* and its transpose, the symmetric connectivity importance between each brain region and others is obtained. Then, the total connection importance score (denoted by *S*) of each brain region with all others can be calculated by summing each row of elements of the resulting matrix. Accordingly, the brain regions corresponding to the top 10 highest scores in the averaged *S* across all test samples are the 10 most important ones for the MDD classification task.

### Comparison results

To evaluate the performance of our EC-GCL framework, we compared it with eight well-known machine learning methods. First, we compared the proposed method with two traditional machine learning methods: Support Vector Machine (SVM) and Multilayer Perceptron (MLP). For these two methods, the upper triangle of the FC matrix was flattened into a 6670 dimensional vector, which was then used as input for the SVM or MLP classifier. We also compared our framework with two well-known GNN models: Graph Convolutional Networks (GCNs) [16] and the Graph Attention Network (GAT) [17]. In addition, our method was also compared with methods designed for fMRI network analysis: BrainNetCNN [18], BrainGNN [19], HI-GCN [20], and A-GCL [21]. For these four methods, we followed the parameter settings described in their original papers to ensure fairness.

Model performance was evaluated by three standard metrics: area under the curve (AUC), accuracy, and F1 score. To ensure robustness, five independent experiments were conducted with five randomly initialized seeds, and the mean and standard deviation of the results are reported in Table 2. The experimental results demonstrate that the proposed EC-GCL framework consistently achieves superior performance across all evaluation metrics on the dataset. These results provide strong evidence for the effectiveness of the EC-GCL model in the classification of MDD.

**Table 2:** Comparison with different algorithms.

Methods	AUC	Accuracy	F1
SVM	66.8±2.9	62.5±3.8	64.1±2.8
MLP	64.4±3.1	63.1±3.0	62.8±2.9
GCN [16]	66.2±2.6	62.5±3.3	64.4±4.1
GAT [17]	66.5±2.7	63.5±3.6	64.8±3.2
BrainNetCNN [18]	68.3±2.8	64.2±3.8	65.8±2.6
BrainGNN [19]	69.0±2.3	67.7±3.6	65.7±3.0
HI-GCN [20]	67.9±3.1	64.5±2.8	65.1±2.5
A-GCL [21]	67.4±2.4	65.9±3.7	64.5±3.5
EC-GCL(Ours)	**71.2±2.1**	**67.8±4.2**	**69.1±3.3**

### Ablation studies

To understand how different parts of our method affect performance, we carried out ablation experiments. We compared the complete EC-GCL framework against three simplified versions:

1. GC-GCL (Graph Convolution based Graph Contrastive Learning): replaces the edge convolution layer with a standard GCN to validate the efficacy of the edge convolution.
2. EC-GCL-RA (Random Augmentation): substitutes the learnable data augmentation module with random edge deletion, showing the importance of adaptive augmentation.
3. EC-GCL-DT (Decoupled Training): separates contrastive learning and supervised classification into two stages instead of joint training, highlighting the effect of joint training.

The results of the ablation experiment are shown in Table 3. The EC-GCL achieves excellent performance in various evaluation indicators compared to the others. This indicates that these strategies using edge convolution, learnable data augmentation, and joint training combining contrastive loss with classification loss, yield substantial improvements in model performance.

**Table 3:** Ablation experiment results.

Methods	AUC	Accuracy	F1
GC-GCL	67.9±2.7	65.9±3.5	67.2±3.2
EC-GCL-RA	67.0±2.9	66.6±3.3	66.2±4.0
EC-GCL-DT	69.1±2.2	**68.2±4.0**	68.4±3.8
EC-GCL	**71.2±2.1**	67.8±4.2	**69.1±3.3**

### Leave-one-site-out experiments

To verify the generalization capability of the proposed method across multi-center data, we used a Leave-One-Site-Out Cross-Validation (LOSOCV) approach. For each validation fold, the data from one site was set aside as the test set, while the data from all remaining sites was used for training. This process was repeated for all sites, meaning the number of folds (*k*) equals the number of sites. For the REST-meta-MDD dataset, this resulted in 10 folds, since it includes data from 10 sites.

To evaluate the overall performance on all sites, we calculated three indicators with the weighted average rather than a simple arithmetic mean. Because the number of samples varies greatly across sites, simply averaging the results of all sites would lead to a disproportionate bias to smaller sites, which are often less representative. To address this, we used the weighted average metrics, with weights proportional to each site’s sample size, which provides a fairer and more reliable measure of overall performance.

**Fig 5:**
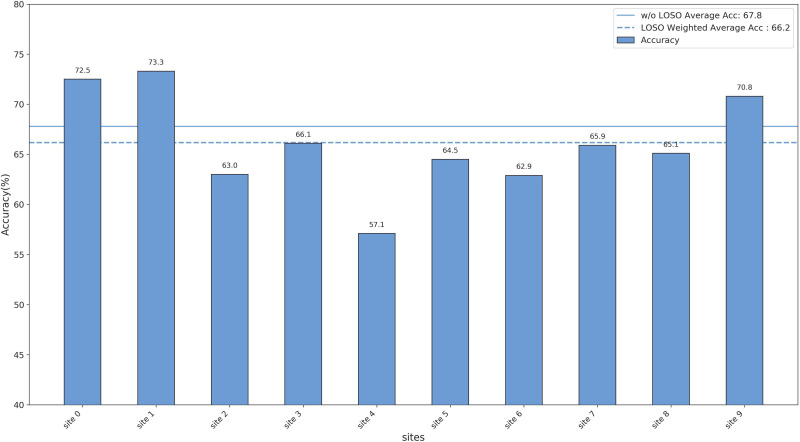
The results of the leave-one-site-out experiments (Accuracy).

**Fig 6:**
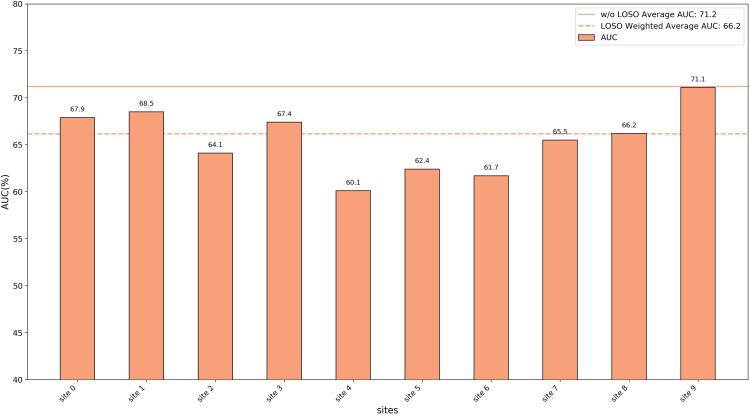
The results of the leave-one-site-out experiments (AUC).

**Fig 7:**
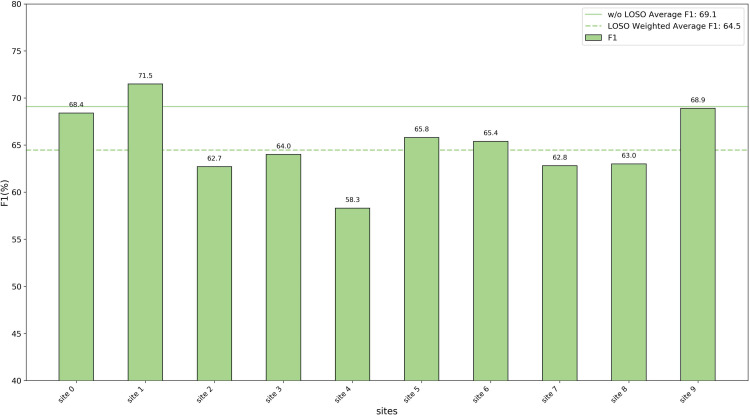
The results of the leave-one-site-out experiments (F1).

We reported the threeevaluation metrics for each site and compared the average results under two settings: LOSOCV and conventional cross validation without LOSO. The detailed results are shown in Figs 5–7. In the REST-Meta-MDD dataset, the weighted average accuracy for LOSOCV was 66.2%, only 1.6% lower than the 67.8% achieved with conventional cross validation. This small difference indicates that the model remains stable even when accounting for differences between sites. Moreover, the experiments confirm that the EC-GCL model is effective at identifying MDD using fMRI brain data from various research centers.

### Interpretability analysis

**Fig 8:**
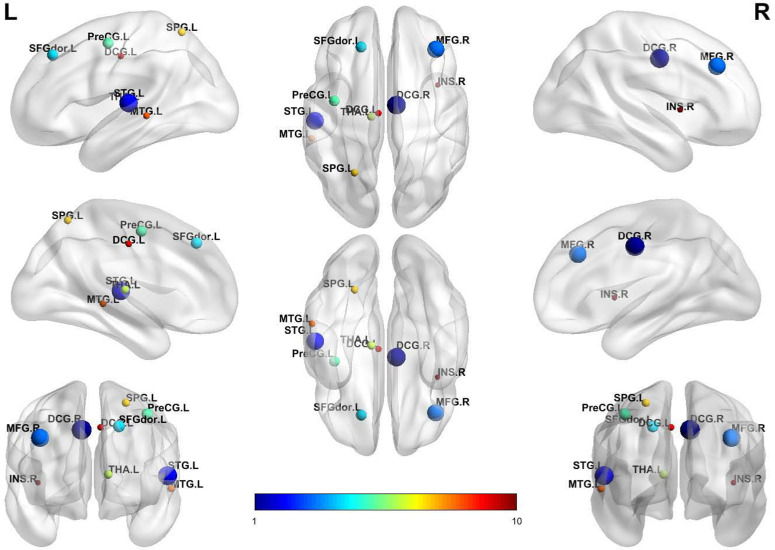
The ten brain regions that have the greatest influence on classification.

From the interpretability analysis, we identified ten brain regions that most strongly influenced the classification results: the right middle frontal gyrus (MFG.R), right dorsal cingulate gyrus (DCG.R), right insula (INS.R), left dorsolateral superior frontal gyrus (SFGdor.L), left precentral gyrus (PreCG.L), left superior parietal gyrus (SPG.L), left superior temporal gyrus (STG.L), left middle temporal gyrus (MTG.L), left dorsal cingulate gyrus (DCG.L), and left thalamus (THA.L). Most of these regions are located on the left side of the brain. The detailed distribution is shown in Fig 8.

## Discussion

This study proposes an intelligent diagnostic framework for MDD that leverages the graph contrastive learning integrating edge convolutional operations with learnable graph augmentation. Relative to conventional GNNs, the EC-GCL framework exhibits enhanced capacity to preserve the spatial specificity of brain regions, thereby improving both diagnostic accuracy and interpretability.

Three key interconnected innovations collectively drive the superior performance of EC-GCL. First, edge convolution replaces traditional message-passing mechanisms to more effectively capture physiologically meaningful connectivity patterns. By processing row and column dimensions of the FC matrix separately, it retains the unique functional roles of individual brain regions in neural circuits. Our ablation experiments have confirmed that replacing standard GCN (GC-GCL) with edge convolution improves AUC by 3.3% (from 67.9% to 71.2%), highlighting the critical role of this design in capturing disease-relevant neural patterns. Second, learnable graph augmentation adaptively discards redundant edges while preserving clinically relevant connections, avoiding the blindness of random augmentation. Compared to random augmentation (EC-GCL-RA), our approach improved AUC by 4.2% (from 67.0% to 71.2%), demonstrating that adaptive augmentation strengthens feature discriminability and reduces overfitting. Third, joint training of contrastive loss, classification loss, and regularization loss ensures the model learns discriminative and invariant features, reducing overfitting and improving generalization. Contrastive loss enforces invariant feature learning between original and augmented graphs, while classification loss anchors the model to diagnostic labels. Regularization loss encourages meaningful edge deletion, further focusing the model on critical connections. Decoupled training (EC-GCL-DT) separates these objectives, leading to a 2.1% AUC reduction, confirming that joint optimization enhances both performance and generalization.

These innovations build on and extend prior advances in graph contrastive learning (. Lin et al. [21] presented graph data augmentation from a spectral perspective by exploring the invariance properties of graphs in the spectral domain. Cai et al. [22] developed a LightGCL with a contrastive augmentation method based on singular value decomposition, which can optimize the graph structure according to global relationships. Consistent with these works, our results validate that structure-aware graph augmentation outperforms random strategies, particularly in clinical datasets where meaningful connections are sparse. However, the graph augmentation of both works employed rule-based perturbations rather than a learnable augmentation module, lacking adaptive optimization tailored to dataset-specific characteristics. Yin et al. [23] advanced an AutoGCL by learning a probability distribution-based graph augmenter in the GCL framework, which can greatly retain the representative structure of the original graph while introducing sufficient variance into the augmented views. Suresh et al. [24] introduced an adversarial GCL (AD-GCL) with trainable edge-dropping graph augmentation, which avoids capturing redundant graph features. Zhang et al. [25] further proposed dynamic memory banks for negative sample expansion. Although these two studies align with our work in addressing the blindness of traditional random graph augmentation via learning-based adaptive strategies, and in integrating augmentation with contrastive learning to strengthen feature discriminability, they adopt the traditional GNN as encoders, lacking directional connectivity capture. By integrating learnable augmentation with edge convolution, our framework enhances both feature specificity and data utility for graph-based MDD diagnosis.

Beyond validating the framework’s diagnostic efficacy, the interpretability analysis enabled by our learnable graph augmentation module further uncovers MDD’s neural mechanisms. By quantifying the importance of retained functional connections via edge-dropping mask analysis, we identified ten specific brain regions that contribute to the classification of MDD. These regions are largely consistent with past research and support core ideas regarding the neural mechanisms of MDD, such as emotion regulation deficits, cognitive impairment, interoceptive dysfunction, and disturbances in sensory-emotional integration [11].

The lateral habenula (LHb), a subregion of the thalamus (THA), is recognized as a key brain region in the pathogenesis of depression [26]. The interaction pattern between specific brain cells (glial cells and neurons) within the LHb serves as a critical driver of depression. When LHb neurons exhibit abnormal firing, they block the brain’s reward and pleasure centers, which in turn induces depressive symptoms including persistent low mood and anhedonia [27]. Ketamine works quickly as an antidepressant by precisely blocking abnormal signals originating from the LHb region of the brain [28]. Additionlly, recent studies have found a decrease in the volume of the left thalamus [3] (THA.L) and a reduction in white matter fiber tract markers for MDD patients [4]. Consistent with these prior findings, our current study also highlights THA.L as a critical region for the identification of MDD. Besides, the dorsolateral superior frontal gyrus (SFGdor.L) and THA.L are integral components of the cortico-striatal-pallidal-thalamic circuit, which exhibits hyperactivation in response to negative stimuli among individuals with MDD [29]. Our results also show that the SFGdor.L is an important region for MDD recognition. Collectively, these results reinforce the notion that changes in neural circuit activity are reliable biomarkers for MDD identification.

The dorsal cingulate gyrus (DCG) acts as the main center for emotion cognition regulation, with a primary role in processing self-referential negative information and emotional conflict. In MDD patients, this region exhibits hyperactivation during episodes of negative self-reflection [30], which weakens control over negative thoughts, leads to repetitive self-focus (rumination), and makes it hard to stop negative thinking. Consistent with the prior research, our study identifies that both the left and right sides of the DCG (DCG.L and DCG.R) are critical biomarkers for MDD.

The insula is also considered a key indicator for MDD due to its role in controlling for mood and the altered brain connections found in patients [31]. Specifically, some studies indicate that before treatment, activity in the right anterior insula may help predict whether an MDD patient will respond better to therapy or medication [32]. Additionally, a reduced volume of the right anterior insula has also been associated with a higher risk of relapse, even after controlling other risk factors such as stressful life events [33]. In MDD patients, the insula shows altered functional connections with other brain regions, including the amygdala and prefrontal cortex [34]. Our study further confirms that the right insula (INS.R) is important for identifying MDD, which matches earlier neuroscience works.

Despite the EC-GCL improves the classification and interpretability for MDD, this study has several limitations that should be addressed in future research. First, the dataset is derived exclusively from Chinese populations, which may limit the generalizability of the model to other ethnic groups. Future work will expand the dataset to include multi-ethnic samples. Second, we relied solely on static rs-fMRI functional connectivity data, ignoring the dynamic nature of brain networks and the complementary information from other modalities. Drawing insights from recent relevant studies [35,36], in the future, we plan to deepen our research in two directions: first, inspired by [35], we will integrate rs-fMRI with clinical data, structural MRI, and other modalities to capture more comprehensive MDD-related information; second, building on [36], we will incorporate dynamic graph learning to model time-varying functional connections, complementing our static graph learning.

## Conclusion

To improve the intelligent diagnosis of MDD, we proposed a new learning framework that uses edge convolution and graph contrastive learning. The framework effectively handles the contradiction between GNN’s permutation invariance and brain region specificity through edge convolution blocks, while the learnable graph augmentation strategy helps retain important functional connections. The results showed that our method outperformed various comparative methods, including traditional machine learning, classic GNNs, and state-of-the-art fMRI analysis models. Additionally, the framework enables interpretability analysis by identifying critical brain regions via retained edges, offering insights into the neural mechanism of MDD.
